# Treatment-resistant depression and major depression with suicide risk—The cost of illness and burden of disease

**DOI:** 10.3389/fpubh.2022.898491

**Published:** 2022-08-11

**Authors:** Rute Dinis Sousa, Miguel Gouveia, Catarina Nunes da Silva, Ana Maria Rodrigues, Graça Cardoso, Ana Filipa Antunes, Helena Canhao, José Miguel Caldas de Almeida

**Affiliations:** ^1^Comprehensive Health Research Centre, NOVA Medical School, Universidade Nova de Lisboa, Lisboa, Portugal; ^2^NOVA Medical School, Universidade Nova de Lisboa, Lisboa, Portugal; ^3^Episaúde – Associação Científica, Évora, Portugal; ^4^Católica Lisbon School of Business and Economics, Universidade Católica Portuguesa, Lisboa, Portugal; ^5^Comprehensive Health Research Centre, Lisbon Institute of Global Mental Health, Lisbon, Portugal; ^6^Department of Mental Health, NOVA Medical School, Nova University of Lisbon, Lisbon, Portugal; ^7^National School of Public Health, UNL, Lisboa, Portugal

**Keywords:** treatment-resistant depression, major depression with suicide risk, cost of illness, burden of disease, disability-adjusted life years (DALYs) lost

## Abstract

**Introduction:**

Treatment-Resistant Depression (TRD) and Major Depression with Suicide Risk (MDSR) are types of depression with relevant effects on the health of the population and a potentially significant economic impact. This study estimates the burden of disease and the costs of illness attributed to Treatment-Resistant Depression and Major Depression with Suicide Risk in Portugal.

**Methods:**

The disease burden for adults was quantified in 2017 using the Disability-Adjusted Life Years (DALYs) lost. Direct costs related to the health care system and indirect costs were estimated for 2017, with indirect costs resulting from the reduction in productivity. Estimates were based on multiple sources of information, including the National Epidemiological Study on Mental Health, the Hospital Morbidity Database, data from the Portuguese National Statistics Institute on population and causes of death, official data on wages, statistics on the pharmaceutical market, and qualified opinions of experts.

**Results:**

The estimated prevalence of TRD, MDSR, and both types of depression combined was 79.4 thousand, 52.5 thousand, and 11.3 thousand patients, respectively. The disease burden (DALY) due to the disability generated by TRD alone, MDSR alone, and the joint prevalence was 25.2 thousand, 21 thousand, and 4.5 thousand, respectively, totaling 50.7 thousand DALYs. The disease burden due to premature death by suicide was 15.6 thousand DALYs. The estimated total disease burden was 66.3 thousand DALYs. In 2017, the annual direct costs with TRD and MDSR were estimated at € 30.8 million, with the most important components being medical appointments and medication. The estimated indirect costs were much higher than the direct costs. Adding work productivity losses due to reduced employment, absenteeism, presenteeism, and premature death, a total cost of € 1.1 billion was obtained.

**Conclusions:**

Although TRD and MDSR represent relatively small direct costs for the health system, they have a relevant disease burden and extremely substantial productivity costs for the Portuguese economy and society, making TRD and MDSR priority areas for achieving health gains.

## Background

Depressive disorders, which can be lasting or recurrent, are characterized by sadness, loss of interest or pleasure, feelings of guilt or low self-esteem, disturbances in sleep or appetite, feeling tired, and low concentration (1). Depression is associated with deficits in individuals' professional, social, and personal functioning, contributing to decreased patients' quality of life. In its most severe form, the depressive disorder can lead to suicide (1, 2).

Globally, it is estimated that more than 300 million individuals, equivalent to 4.4% of the world population, are affected by depression (1). A recent study based on Global Burden of Disease (GBD) estimates on subjects aged 10–24 years, shows that Portugal has the highest prevalence of mental disorders in Europe during the period 1990–2019. It showed also that YLDs due to mental disorders are the first cause of disability at this age in Portugal, as in other European countries (3). Portugal is the second country in Europe with the highest prevalence of psychiatric diseases, and mood disorders had a prevalence of 7.9% in 2009 (4).

Although several drugs are indicated for the treatment of depression, studies reveal that one to two-thirds of patients will not respond to the first prescription, and 15–33% will fail to respond to multiple interventions (5).

According to the literature, there is no exact definition for treatment-resistant depression (6). However, it has been established that the term refers to an inadequate response to at least one antidepressant, with adequate dose and duration, in patients with depression (7). Nevertheless, what constitutes an inadequate response is still the subject of debate. Nowadays, for many specialists, the goal of treatment is to achieve remission (8). Despite the lack of consensus, the duration of response assessment is generally defined as a minimum of 6 weeks of treatment (9).

There are few estimates of the prevalence of treatment-resistant depression. Available data points to a global life-time prevalence of Major Depression (MD) of 10 to 15%, and some studies estimate this prevalence to be between 8.1 and 11.2% in low/medium income countries and 13% in high-income countries (10, 11). The epidemiology of Treatment-Resistant Depression's (TRD) is not so well studied and characterized due to the heterogeneity of criteria and methodologies and scarcity of epidemiology-related studies. However, it is estimated that more than one-third of treated patients with MD progress to TRD (7, 11, 12), a proportion that has been corroborated by the study “Sequenced Treatment Alternatives to Relieve Depression” (study STAR^*^D), one of the largest and most important studies that evaluated the burden of treatment-resistant depression (13). There has been an increase in the number of people diagnosed with this type of depression. These patients tend to have higher comorbidities with other psychiatric disorders, incapacity for work, absenteeism, more frequent hospitalizations, which consequently generates higher costs for the health system (14).

Much of the cost and disability associated with depression are explained by resistance to treatment (15). Depressive disorders are responsible for an overall loss of more than 50 million years of life adjusted for disability (1). Estimates indicate that depression will be the leading cause of global disease burden worldwide by the year 2030 (1, 2, 16).

Management of treatment-resistant depression requires a multimodal approach, which includes pharmacological and non-pharmacological intervention. It may include cognitive-behavioral therapy, electroconvulsive therapy, vagus nerve stimulation, and transcranial magnetic stimulation (12, 14, 17–20). However, pharmacological therapy remains the main component of treatment (14).

Suicidal ideation can occur in several psychopathological contexts, namely in depressive pathology (21). In the pharmacological treatment of patients with depression with suicide risk, the drugs must have a rapid and early effect. Even in patients who respond to conventional antidepressant medication, obtaining a response always implies a latency time of action that can take up to 3 weeks after treatment initiation. This delay can be fatal in severe cases, hence the need for therapeutic alternatives with a rapid onset of action, which justifies the evaluation and therapeutic indication of specific drugs for this purpose.

The endeavor of the present study is to contribute to a comprehensive perspective of the costs endured by the Portuguese health system, generated by TRD and MDSR.

Our research on the burden and costs of TRD and MDSR in Portugal had two main objectives. The first was to uncover the disease burden by estimating the component attributed to treatment-resistant depression and major depression with suicide risk based on the following indicators: mortality, morbidity, and Disability-Adjusted Life Years (DALYs) for the year 2017. Our second objective was to estimate the economic cost of diagnosing and treating TRD and MDSR, including direct costs (medical and non-medical costs) and indirect costs (loss of productivity—relevant to the perspective of society) for the year 2017.

## Methods

The primary source of the epidemiological information used in this study is the “National Epidemiological Study of Mental Health” (NESMH) (4), which took place between 2008 and 2009. This study is the most accurate and robust national data available, assessing primary data collected in a representative sample of the adult population in mainland Portugal. The results of the NESMH were adjusted in our study regarding the demographic composition of the population due to aging, and all estimates were calculated using the population and demographic structure of the year 2017. Portugal's NESMH was part of the World Mental Health Surveys Initiative (WMHSI), promoted by the World Health Organization and Harvard University. WMHSI was coordinated internationally by Prof. Ronald Kessler and is fully described elsewhere (22–27). The methodology and implementation of NESMH are described in Xavier et al. (16). The NESMH questionnaire (16) was divided into two parts to reduce the time taken to answer. Part I used a total sample number of 3,849 participants to represent the general population and included an initial baseline assessment to diagnose major mental disorders. Part II of the questionnaire was answered by 2,060 individuals which included all participants with a mental disorder diagnosis and a random sample of 25% of participants without psychiatric disorder. Two weights were created to accommodate the stratification of the sample. The data in Part I were weighted for the differential probability of selection (between and within households), non-response bias, and discrepancies between the sample and the geographic and sociodemographic distribution of the Portuguese population assessed in the *census*. The data of Part II was additionally weighted for the differential sampling of participants from Part I to Part II.

Psychiatric disorders were assessed using the World Health Organization World Mental Health Composite International Diagnostic Interview version 3.0 (WMH-CIDI 3.0), a comprehensive and fully structured interview designed to assess mental disorders according to the definitions and criteria of the DSM-IV (28) and ICD-10 (29).

### Estimates of the prevalence of treatment-resistant depression

Before obtaining epidemiological information related to TRD, the concept of TRD was operationalized within the framework of available epidemiological data, adopting a “proxy” as collected data does not strictly respect any of the possible definitions of TRD. The criteria used to define a TRD case included the presence of a major depressive disorder in the last 12 months assessed objectively with a validated scale, and a negative answer to the question “Have you ever received treatment that you considered to be useful or effective?”, present in the depression module of WMH-CIDI 3.0.

Results show that the global prevalence rate of treatment-resistant depression in the population aged 18 and over in Portugal should be 1.1%. This prevalence corresponds to about 14.9% of the population with major depressive disorder. These values are obtained with a small number of observations (*n* = 43) in a representative sample of the population, using the previously described weights. Combining this prevalence rate, estimated using the referred weights, with the estimates of the Mainland's adult population in 2017 from Portugal's National Institute of Statistics (INE), a total prevalence for TRD of ~90 thousand patients is obtained.

### Estimates of the prevalence of major depression with suicide risk

In the case of MDSR, the operationalization of the concept was achieved by combining major depressive disorder with suicidal ideation. Both situations were reported in the 12 months before the moment of the survey. The results obtained indicate that the prevalence of MDSR is expected to be around 0.8% of the adult population in Mainland Portugal. Multiplying the Mainland adult population by this rate, a total prevalence of MDSR of ~64 thousand patients was obtained in 2017.

### Estimates of combined prevalence of treatment-resistant depression and major depression with suicide risk

To fully understand the data presented above, it should be highlighted that some patients check both criteria for identifying the different types of depressive pathology and, therefore, appear in both prevalence estimates. This reality reinforces the adequacy and need to study the cost and burden of the two clinical entities together.

The crossing of individual information in the survey with objective and representative data from the Portuguese population shows that about 11.2% of patients with TRD also suffer from MDSR. Alternatively, it appears that about 19.3% of patients with MDSR suffer from TRD. This information implies that the characterization of these patient populations should consider the prevalence of TRD without MDSR, the prevalence of MDSR without TRD, and the joint prevalence of TRD and MDSR. The aggregate results present a combined prevalence of Treatment-Resistant Depression and Major Depression with Suicide Risk of 11,283 patients who simultaneously meet the criteria for both types of depression (7.9% of the total study patients).

### Severity level distribution

The National Epidemiological Study on Mental Health (NESMH) contains information on the severity levels of the disease. The classification of patients by severity levels was based on the criteria adopted in the World Mental Health Survey (WMHS) (see the paper by Xavier et al.). For patients with TRD and MDSR, the distribution estimated by levels of severity can be seen in Table 1.

**Table 1:** Distribution of estimated TRD and MDSR cases by the level of severity.

**Severity level**	**TRD**	**MDSR**
Severe cases	27.7%	64.4%
Moderate cases	60.7%	31.0%
Mild cases	11.6%	4.6%

TRD, Treatment-resistant depression; MDSR, Major Depression with suicide risk.

Table 1 shows that the distribution by severity levels indicates a difference between TRD and MDSR, with the latter pathology having significantly higher levels. In the case of the joint prevalence of TRD and MDSR, it is assumed that the distribution by severity levels is that of the MDSR, as it is the most severe condition.

### The matter of duration

An important component necessary to estimate the burden of TRD and MDSR is the duration of the episodes and, consequently, the fraction of the time affected by the disease in the reference year for the analysis. The distribution of the duration of episodes of major depression appears to be very heterogeneous. Spijker et al. (30) retrospectively used data from the Dutch mental health survey to estimate the duration of an episode of major depression. They found that in 50% of cases the duration was 3 months or less, in 63% of cases was 6 months or less, in 76% of cases was 12 months or less, but in 20% of cases the duration was found to be 24 months or more. Spijker et al. (30) reported that the median duration was 3 months, but the estimated average duration was 8.4 months. No specific references were found for the case of TRD and MDSR. In the present study, the assumption is made that the duration of TRD and MDSR can be approximated by the duration of episodes of major depression. The study by Ferrari et al. (31), which synthesizes information on estimates of the various parameters necessary to calculate the disease burden, indicated that the average duration used in the Global Burden of Disease, resulting from a synthesis of the literature, was 37.7 weeks. The present study has used this value, i.e., it is assumed that depression affects the health of patients at an average of 72.3% of the time in the year in which an episode occurs.

#### Burden of disease

The burden of disease was estimated through the Disability-Adjusted Life Years (DALYs). The most recent version of the methodology introduced by the World Bank and the World Health Organization (WHO) was adopted in this study (32).

DALYs are a measure, expressed in time, of the amount of health lost due to the disability generated by disease or premature death. The measure includes two indicators: (1) the years lost due to premature death (Years of Life Lost - YLL), the lost time being operationalized as the difference between age at the time of death and the standard life expectancy for that age; and, (2) the Years lived with Disability (YLD), where the time spent suffering a disability is considered (33).

The equation used to estimate the number of DALYs lost by an individual is as follows:


$$\begin{array}{l} DALY\left(c,s,a,t\right)=YLL\left(c,s,a,t\right)+YLD\left(c,s,a,t\right) \end{array}$$

Where *c* is cause, *s* is sex, *a* is age, and *t* is time.

Disability is measured by a coefficient with values between 0 (without any disability, perfect health) and 1 (total disability or death). Standard life expectancy results from a reference mortality table designed to have universal applicability.

### Years of life lost due to premature death

The years of life lost due to premature death (YLL) are calculated by multiplying the number of deaths caused by the disease under analysis and the years of life lost, which are a function of the age at which death occurs. In the case of depression, it is not usually taken as a direct cause of death. However, a significant fraction of suicide deaths can be statistically attributed to depression. In this context, YLL were estimated considering that a fraction of the overall suicide mortality is attributable to depression. Following the approach that uses the concept of attributable fraction (34), the fraction of total mortality attributable to MDSR was determined by the equation:


$$\begin{array}{l} Attributable\;Fraction\;\left(AF\right)=\;\frac{p\left(RR-1\right)}{p\left(RR-1\right)+1} \end{array}$$

where RR is the Relative Risk of death in patients with the disease under study and p is the prevalence of the disease. The RR of suicide mortality in major depression considered by Ferrari et al. (31) was 19.9. In the present study, TRD, and especially MDSR, would be expected to have higher RRs than those of major depression in general. In the literature, specific estimates were not found. The adopted methodology was to estimate the attributable fraction of suicides to major depression and, later, through the opinion of experts to obtain the proportion of this attributable fraction (AF) that applies to the two types of depressive pathology under study.

According to NESMH, the prevalence of major depression in the year prior to the survey interview was 6.8% (4). The AF that results from the above equation is therefore 56.24%.

The next step was to estimate the proportion of these suicides attributable to the prevalence of TRD and MDSR. Our research assumed that, as in these data, suicide did occur, so ex post it is a case of MDSR. Considering the diagnostic criteria for major depressive disorder in its different configurations, the possibility of some suicides occurring in patients who would not be diagnosed with MDSR should be considered. Bearing this is mind, it was conservatively assumed that 90% of suicides attributable to major depression in general can be more specifically attributed to MDSR or TRD. Assuming this rate of 90% (out of the previous AF of 56.24%), the final attributable fraction is 50.6%. This is the percentage of the disease burden and costs generated by suicide deaths that will be attributed to MDSR and TRD in the present study.

### Years lived with disability

DALY indicator, as a metric of disease burden, estimates, in addition to mortality, the disease burden generated by morbidity, considering that the time lived with a disease contributes to the years of life lost as that such a disease is disabling. The equation used to estimate the YLD number is as follows:


$$\begin{array}{l} YLD\left(c,s,a,t\right)=P\left(c,s,a,t\right)\times DW\left(c,s,a\right) \end{array}$$

Where P is Prevalence of cause (c), by age (a) and sex (s), in year (t); and DW is Disability Weight specific to the cause (c), age (a) and sex (s).

The disease burden was estimated from the indicators: prevalence, mortality, disease duration.

The estimation of YLDs requires the use of disease-specific weighting or disability coefficients and is calibrated according to the different levels of disease severity. The most current version of the weights was published by Salomon et al. (35) and the weights by severity level for the case of depression are shown in Table 1.

The use of these weights in the case of TRD and MDSR depends on the distribution of patients by severity levels. This information, from NESMH is shown in Table 1, and can be reviewed in columns (3) and (4) of Table 2. The average values of the weights are very high compared to other pathologies, probably because an expressive proportion of patients with major depression are not actually being treated, which in the case of MDSR is reinforced by the fact that even in the target patients of treatment they do not evaluate it as being effective.

**Table 2:** Disability weights considered in the burden of disease estimates.

**Severity level** **(1)**	**Disability weight** **(2)**	**Proportion in the prevalence of TRD** **(3)**	**Proportion in the prevalence of MDSR** **(4)**
Mild	0.1451	11.6%	4.6%
Moderate	0.396	60.7%	31%
Severe	0.658	27.7%	64.4%
Average weight	–	0.439	0.553

Sources: Salomon et al. (35) regarding (2), and experts' qualified opinions and author's calculations to (3) and (4).

### Costs of illness

#### Direct costs

The direct costs of TRD and MDSR resemble the monetary appreciation of the resources consumed in treating these diseases. The study of direct costs is based on the previously presented estimates of disease prevalence and the information on the pattern of use of resources contained in the “National Epidemiological Study of Mental Health” (NESMH) (4) and in the opinion of their experts. Microdata available in the 2017 Hospital Morbidity Database regarding inpatient and outpatient episodes registered using the International Classification of Diseases, tenth version (ICD-10 CM) and billing in Homogeneous Diagnostic Groups (HDG), were considered. Microdata was used to estimate the number of relevant hospitalizations and outpatient episodes, as well as the respective costs. The study also used aggregated data on the consumption of drugs associated with the treatment of depression, from IQVIA and hmR, and expert opinions were used to estimate costs in areas where databases or other quantifiable sources of information are not known officially or academically recognized. Finally, in this study, the unit costs of hospitalizations, hospital consultations, and complementary means of diagnosis and therapy were obtained from the prices defined in Portuguese Law (Ordinance No. 207/2017, of 11 July).

##### Costs of hospitalizations and ambulatory hospital visits

Estimates of hospital activity related to hospitalization episodes associated with TRD and MDSR are reported. The estimates are based on an analysis of the Hospital Morbidity Database for 2017. In this database, the use of ICD 10 hinders a finer analysis with the separation of TRD and MDSR. Thus, for greater accuracy, the hospitalization episodes associated with TRD and MDSR are presented together.

Registered and coded episodes are included in this database, including coded and registered hospital outpatient episodes. The identification of relevant episodes associated with TRD and MDSR was based on the International Classification of Diseases. The selection of relevant cases was made by the clinical team and experts. The episodes on which the subsequent analysis is based have all been classified with the ICD 10 CM. The use of the ICD 10 - CM classification was evaluated to guarantee the compatibility between the selected episodes and the diseases under study. The starting point was given by the ICD 10 - CM encodings. Major depressive pathology, single episode, and ICD 10 - CM. 0-9 Major, recurrent, depressive pathology. It was also necessary to add some episodes that were considered to be relevant and that were not part of the preliminary analysis. Thus, a set was added to the selected episodes in which the main diagnosis was “suicidal ideation” (ICD 10-CM R45851) provided that secondary diagnoses (from d2 to d50) were included or a sub-item of diagnoses F32 (Pathology major depression, single episode), or a sub-item of diagnoses F33 (major, recurrent depressive pathology). The use of episodes was further refined by considering additional information on the GDHs of the selected episodes.

##### Costs of pharmacological therapy

Estimates of drug costs in the treatment of TRD and MDSR are based on the intersection information from the EENSM regarding the consumption of medicines, information on the drug market from IQVIA and hmR, and finally, information on the drug market required selection and quantification criteria designed by experts.

The following classes of drugs were studied, following the terminology of the Anatomical Therapeutical Chemicals classification (ATC): N6A Antidepressants and Mood Stabilizers, N5A Antipsychotics, N5C Tranquilizers/Anxiolytics, and N5B Hypnotics/Sedatives.

Detailed data were obtained at the level of pills or equivalent since it was not possible to have access to market statistics specifying the quantities based on Defined Daily Doses (DDD). The available data made it necessary to make assumptions about the average consumption for each class of drugs. Average consumption patterns of one, two, or three tablets per day were chosen, depending on the class of medication.

##### Costs with complementary means of diagnosis and therapeutics

According to the experts, in the context of the usual follow-up, patients with TRD and MDSR tend to do routine tests twice a year. In addition to these consumptions, about 5% of patients undergo thyroid tests. Finally, about 5% of patients are submitted to imaging tests whose main objective is to overlook the possibility of other somatic pathologies. In two-thirds of the cases, the exam is a Computed Axial Tomography (CT) and, in the remaining cases, a Magnetic Resonance Imaging (MRI).

Based on data from NESMH, it is possible to obtain the proportion of patients who had any contact with the health system during the year prior to the study, both in mental health and primary care. According to that information, 26.3% of the patients with TRD had some contact with mental health services, and 39% had some contact with primary health care services. Assuming that the contact probabilities in the two areas are independent, the probability of having at least one contact for patients with TRD is given by 1- (1–0.263) × (1–0.39) = 0.55. Consequently, this result will calibrate the estimates that follow, as it is assumed that only patients in contact with the health system generate consumption of complementary means of diagnosis and therapeutics. Specifically, for the case of patients with TRD (not including joint prevalence with MDSR), the following analysis assumes that the pattern of resource use of complementary means of diagnosis and therapeutics applies to 55% of patients.

In regard to patients with MDSR, estimates based on NESMH indicate that 43.2% will have some contact with mental health services and 57.7% with primary health care services. As in the previous case, independence of the probabilities of contact is assumed, resulting in an estimate of the percentage of patients with some type of contact with the health system of 76%. Weighting the two percentages by the proportion of patients with TRD only (55.5%) and patients with MDSR (44.5%), an average percentage of patients with contact of 64.4% is obtained. Applying this percentage to the prevalence in 143,163 patients results in 92,147 patients who generate a consumption of complementary means of diagnosis and therapeutics. It should be noted that the estimates presented treat the cases in which the patients have TRD and MDSR together as equivalent to those of the patients with the most severe situation, a methodology already adopted in other parts of this study.

We also considered the routine analyses that patients with TRD and MDSR would do twice a year, on average. A second set of analyses, related to the thyroid test, is carried out annually by about 5% of the patients. In addition, about 5% of patients undergo a CT scan (2/3 of the cases) or an MRI (remaining 1/3) to screen for other pathologies. In 50% of these cases, it is necessary to use contrast, increasing costs.

##### Costs of emergency department visits

According to the billing rules of the Portuguese NHS, episodes of urgency followed by hospitalization are integrated into hospitalization prices. It is then assumed that the Homogenous Diagnostic Groups (HDG) hospitalization prices are estimates of the overall costs of hospitalization, including the costs of the previous emergencies that generated these hospitalizations. For this reason, the costs of emergency department visits will estimate only the costs of emergency episodes without hospitalization.

#### Indirect costs

Indirect costs result from the loss of productivity of patients and are defined in the present study as the value of production losses attributed to treatment-resistant depression and major depression with suicide risk. These may include absenteeism as short-term disability, premature exit from the labor market as long-term disability, and productivity lost by premature death.

The sources of information to identify these costs include academic literature, Portuguese databases, observational studies, and surveys conducted in Portugal. Other variables, such as the average wages by sex and age, will be estimated based on data from the 2017 Personnel Tables of the Portuguese Ministry of Labor, Solidarity, and Social Security.

Labor costs, given by gross wages and employers' social security contributions, are the best measure of the productivity of potential workers, following the Human Capital theory. The average salary, by gender and age group, is added by the employer's contribution to Social Security (23.75%). The resulting value is multiplied by 14 to obtain an estimate of annual productivity.

##### Long-term indirect costs: Effects on employment

The performed analysis takes into account employment until the age of 65 to consider a better approximation to the effective age of leaving the labor market. This effective age reflects that not all workers retire at the official retirement age, given the existence of multiple exceptions: the receipt of disability pensions, early retirements after long-term unemployment, and other situations of an early exit from the labor market.

The employment rates of the population with TRD were approximated by the employment rates of the population with Major Depression, and the employment rates in the population with MDSR were approximated by the employment rates in the group of people with suicidal ideation in the last 12 months. Following the principle of considering people with both types of depression have the most serious disease, joint cases of TRD and MDSR were included in the estimates for TRD, as it exhibits a greater impact on employment rates.

To monetize the lost production due to the lower employment levels, the Human Capital approach was employed, and lost production was approximated by the wage costs that workers would receive.

##### Short term indirect costs: Absenteeism and presenteeism

To estimate the daily productivity lost due to absenteeism, the average annual salary was divided by 230, corresponding to the number of working days per year, given that absenteeism, by definition, only occurs on these days.

The next step in estimating the indirect costs of absenteeism and presenteeism is to estimate the employment of patients with TRD and MDSR, which is achieved by combining estimates of disease prevalence and employment rates by gender, age group, and disease used in the previous section. For the reasons previously indicated, patients with TRD and patients with TRD and MDSR are linked together.

A viable way to identify the incremental effect of the diseases under analysis on absenteeism was to consider the difference between the days of absenteeism in the population with the diseases under study and the days of absenteeism in the general population.

*The methodology adopted to estimate the cost of presenteeism assumed that 1 day of presenteeism has a weight of 0.25 days of absenteeism*. There is no single convention on estimating the cost of presenteeism (36). The literature and the sources available do not provide unambiguous estimates. Drummond et al. (37) mention explicitly that “productivity may be lost even though the worker remains at work. This is often called ‘presenteeism' and has been argued to be a major proportion of the productivity lost through mood disorders (p. 248).” A reference in that textbook (38) formulates an idea that justifies giving some attention to presenteeismpresentism in depression: “The relative importance of presenteeism compared with absenteeism in this disease area is likely because individuals with depression or anxiety tend to stay at work and perform suboptimally rather than take sick leave (p. 1148).”

A review from 2017 (39) shows numerous instruments, surveys and evaluation methodologies. One of this methodologies is based on the conversion of presenteeismpresentism days in proportional reductions in productivity compared with absenteeismabsentism days. If one absenteeismabsentism day is the unit, what should be the fraction to inpute to a presenteeismpresentism day? In some surveys in the literature that fraction can be estimated based on the inneficiency rates self-reported by the patients. However, that type of information was not avaliable in our case. Some contributions in the literature allow us to calculate the ratio of days lost to absenteeismabsentism to equivalent days lost due to presenteeismpresentism [examples Smit et al. (40), in a Dutch context; Uribe et al. (41), in a Colombian context]. However, these papers were heterogeneous in their results, which they reported as total days lost and they were not explicit on the ratio that equalized absenteeism days and presenteeismpresentism days. Using our assumption of 0.25 days of absenteeism per presenteeismpresentism day we obtained conservative results but not totally outside the ballparks of the results in the studies mentioned.

Thus, an estimate is obtained for the total effect of each disease measured by additional equivalent days of absenteeism per year.

##### Indirect costs of premature suicide mortality

The indirect costs generated by suicide match the current value of all future production that would have been carried out by the deceased if he/she had survived. The updated rate used in this analysis is 4%, as outlined in the current guidelines for conducting health technology assessment studies in Portugal (42). The convention of estimating future values of employment rates and wages by age and gender according to the statistics for 2017 was followed. The population's probabilities of survival are also used to calculate the expected value of future productivity. It is assumed that patients deceased due to suicide would have the survival probabilities given by the 2016–2018 Mortality Tables for men and women in general (43).

## Results

### Burden of disease

#### Years lived with disability

Using data on prevalence, disability weights, and the fraction of the year corresponding to the duration of the disease, variables, and parameters presented in methods, the estimates of YLD obtained are shown in Table 3.

**Table 3:** Years lived with disability—global results.

	**TRD**	**MDSR**	**TRD + MDSR**	**Total**
Prevalence	79,401	52,479	11,283	143,162
YLD	25,228	20,989	4,513	50,730

TRD, Treatment-resistant depression; MDSR, Major depression with suicide risk; YLD, Years lived with disability; rounding to units.

**Figure 1:**
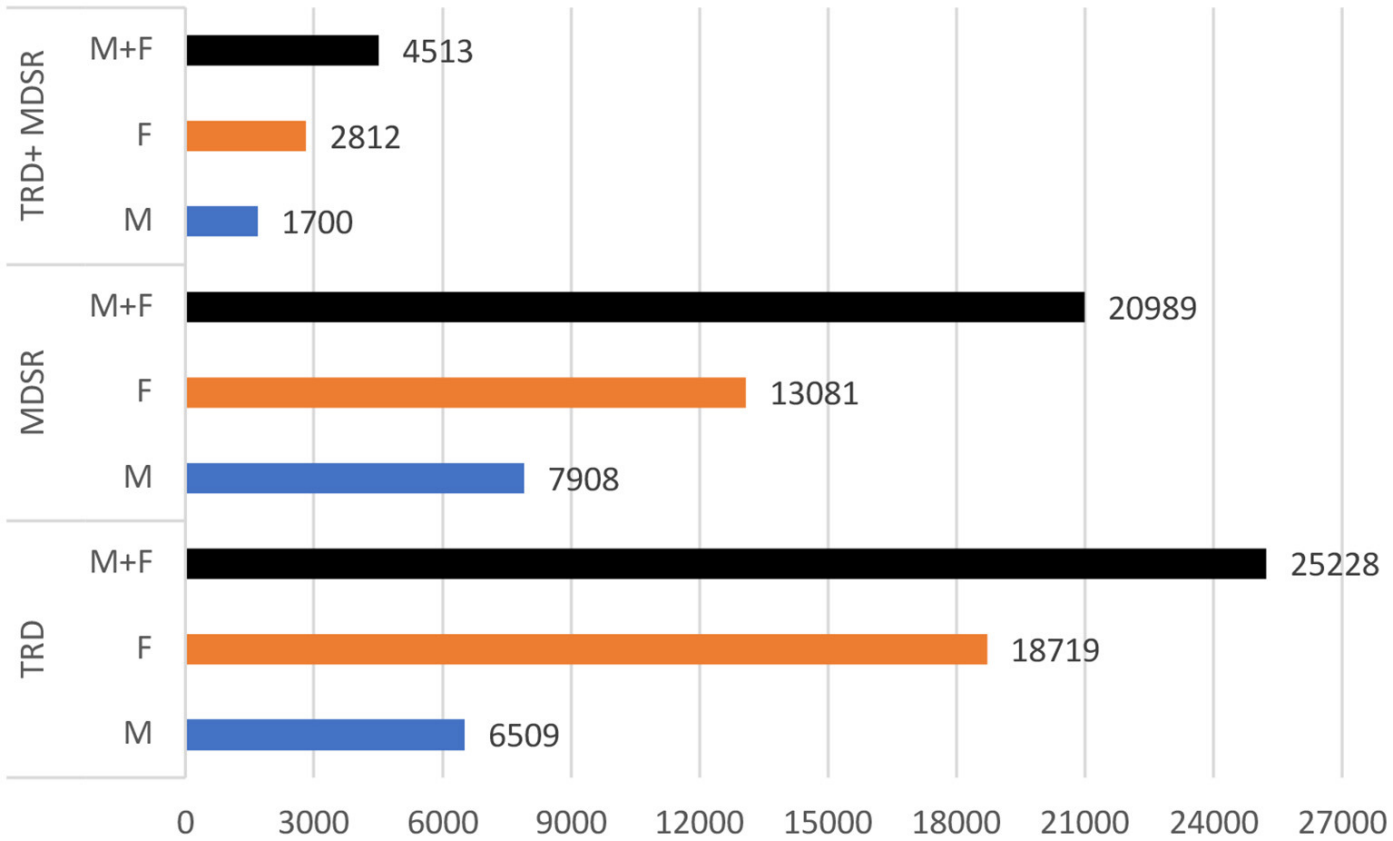
Years lived with disability. TRD, Treatment-resistant depression; MDSR, Major depression with suicide risk; YLD, Years lived with disability; M, Male; F, Female; rounding to units.

The global data of YLD can be broken down by type of depression, sex, and age group. Figure 1 summarizes the detailed information on the years of life lived with disability.

#### Years of life lost due to premature death

YLL are associated with deaths that are officially designated as “Intentionally self-inflicted injuries and sequelae”.

Using a reference mortality table defined by the Global Burden of Disease, almost 31 thousand years of life were prematurely lost due to suicide in 2017, with 73.2% of this total attributed to men. The years of life lost due to suicide (including all ages) constitute 2% of the YLL due to premature mortality, this proportion being 2.5% for men and 1.3% for women.

However, only a fraction of these events is attributed to TRD and MDSR. This topic was studied in the Section Methods, where an attributable fraction of 50.6% was estimated. In total, these estimates are 11,440 YLL for men and 4,187 for women making a total of 15,627 YLL due to premature death attributed to TRD and MDSR.

#### Disability-adjusted life years

Adding years lived with a disability to the years lost due to premature death attributed to TRD and MDSR, separately and together, we obtain the total DALY generated by the two types of depression under study. The results obtained can be seen in Table 4, where the total burden of the disease of TRD and MDSR is measured by the loss of 66,357 years of life adjusted for disability in 2017.

**Table 4:** Years of life lost due to premature death (YLL).

	**Attributable YLL (at YLL)**	**All suicide YLL**	**All YLL**
M	11,440	22,602	903,374
F	4,187	8,272	664,243
Total	15,627	30,874	1,567,617

YLL, Years of Life Lost; M, Male; F, Female.

Although there is an imbalance between men and women in the YLL due to premature death, with men losing 2.7 years for every year lost by women, the opposite is true in the case of YLD (Table 5). Thus, with regards to the results, women have a greater number of YLD than men, corresponding to 58.5% of total years lost.

**Table 5:** Disability-adjusted life years attributable to TRD and MDSR, 2017.

	**YLD**	**YLL**	**DALYs**	**%**
M	16,117	11,440	27,557	41.5%
F	34,613	4,187	38,800	58.5%
M+F	50,730	15,627	66,357	
%	76.4%	23.6%		

YLD, Years lived with disability; YLL, Years of Life Lost due to premature death; DALYs, Disability-adjusted life years; TRD, Treatment-resistant depression; MDSR, Major depression with suicide risk; M, Male; F, Female; rounding to units.

Despite the enormous burden of premature suicide death disease attributed to the two types of depression understudy, the YLL constitute less than a quarter of the disease burden. The high prevalence of TRD and MDSR, and the high levels of disability that these depressive pathologies instigate, make the YLD amounting to more than three times the years lost due to suicide attributed to the depressive disorders under study.

### Cost of illness

#### Direct costs

##### Hospitalization costs

The result of the final analysis led to the identification of 1,696 relevant episodes. A subsequent analysis identified some patients with multiple episodes, so the estimated number of hospitalized patients was 1,502. The total cost of these episodes was estimated at € 3,083,109.

##### Pharmacological therapy costs

The collective costs of medication for TRD in 2017 were estimated to be € 4,244,921. In 2017, the estimates made pointed to a drug expenditure for the MDSR treatment of around € 6.9 million. Globally, the collective expenditure on drugs for the treatment of TRD and MDSR amounts to € 11 million.

##### Costs with complementary means of diagnosis and therapy

The overall results regarding the costs of complementary means of diagnosis and therapy indicated an expense of € 4,049,524 in 2017. An estimated separation of these costs with complementary means of diagnosis and therapy indicates that costs for TRD alone would have been € 1,920,662 and € 2,128,862 for MDSR.

##### Medical appointments costs

The final result obtained is that TRD and MDSR generated 398,511 annual medical visits in 2017. The reference price in Ordinance no. 207/2017, Art. 15, no. 1 is € 31, which applies to ambulatory psychiatry visits and general and family medicine visits, which results in an estimated expense total of € 12,353,851. This amount can be broken down into the cost attributed to TRD (€ 3,913,675) and the cost attributed to MDSR (€ 8,440,176).

##### Emergency episodes costs

An analysis of the 1,696 inpatient episodes studied in the previous sections showed that 799 of these episodes occurred at the emergency room. It is estimated that the number of emergency episodes due to TRD and MDSR in 2017, without hospitalization, was *N* = 799 ^*^ (100-7.94%)/7.94% = 9,264. The average price obtained for an emergency in 2017 was € 88.94. Multiplying this value by the estimated number of emergencies without hospitalization results in a collective cost of € 824,364.

##### Total direct costs

The global results obtained are summarized in Table 6. The global direct costs of TRD and MDSR to health system is 31 million euros. Medical visits (40% of the expenditure calculated) and pharmacological therapy (34%) are the components with the most relevant costs, while the remaining components of direct costs having a substantially less weight.

**Table 6:** Summary of direct costs.

	**Total**	**(%)**
Hospitalizations	3,083,109 €	10.0%
Pharmacological therapy	10,468,895 €	34.0%
Complementary means of diagnosis and therapy	4,049,524 €	13.2%
Medical appointments	12,353,851 €	40.1%
Emergency episodes	824,364 €	2.7%
Total	30,779,743 €	100%

#### Indirect costs

##### Long-term indirect costs—Effects on employment

The overall estimate for the costs of the lowest employment rate in the population with Treatment-Resistant Depression or Major Depression with Suicide Risk is € 834,786,764

##### Short term indirect costs—Absenteeism and presenteeism

Human Capital methodology was used, with daily labor costs to obtain the costs of absenteeism and presenteeism from TRD and MDSR. The global estimate of the costs of absenteeism and incremental presenteeism generated by TRD and MDSR is shown in Table 7. An estimated € 180.3 million is divided into approximately equal parts between men and women. The 35–49 years age group generates a greater fraction of the costs than the other age groups.

**Table 7:** Costs with presenteeism and absenteeism (€), by disease, age group, and sex.

	**TRD** + **TRD and MDSR**		**MDSR**		**Total**
**Age group**	**M**	**F**	**M**	**F**	
<34	5,989,558	16,112,571	26,180,113	17,500,500	65,782,741
35–49	8,566,711	20,760,965	32,607,277	19,636,170	81,571,123
50–64	4,909,932	10,531,366	11,417,642	6,085,475	32,944,415
Total	19,466,201	47,404,902	70,205,032	43,222,144	180,298,279

TRD, Treatment-Resistant Depression; MDSR, Major Depression with Suicide Risk; M, Male; F, Female; rounding to units; results presented in € (euro).

##### Indirect costs of premature mortality due to premature death

Total indirect costs due to premature death attributed to TRD and MDSR is estimated at € 56,604,415. Eighty-one percent of this total (€ 45,615,760) is attributed to men and 19% (€ 10,988,655) to women.

Table 8 shows total Direct and Indirect Costs Attributed to TRD and MDSR: The total indirect costs related to TRD and MDSR collectively reached € 1.1 billion, with men accounting for 36.7% of this cost. According to the type of costs, absenteeism/presenteeism was found to be responsible for 16.8% of the total costs, while the reduction of employment and the costs of premature mortality were responsible for 77.9 and 5.3% of the total indirect costs, respectively.

**Table 8:** Direct and Indirect costs (€) attributable to TRD and MDSR.

**Direct costs**	
Hospitalizations	€ 308,310,900
Pharmacological therapy	€ 1,046,889,500
Complementary means of diagnosis and therapy	€ 404,952,400
Medical appointments	€ 1,235,385,100
Emergency episodes	€ 82,436,400
Total direct costs	€ 3,077,974,300
**Indirect costs**	
Absenteeism and presenteeism	€ 18,029,827,900
Employment reduction	€ 83,478,676,400
Premature death	€ 5,660,441,500
Total indirect costs	€ 107,168,945,800
Total costs	€ 110,246,920,100

TRD, Treatment-Resistant Depression; MDSR, Major Depression with Suicide Risk; rounding to units; results presented in € (euro).

Finally, it should be noted that the direct costs supported by the health system are very small when compared to indirect costs. The direct costs calculated are only 2.7% of the total costs, that is, the sum of all types of estimated costs.

## Discussion

This study focused on estimating the years of life lost attributed to treatment-resistant depression and major depression with suicide risk, the burden of the disease, and the direct and indirect costs of these diseases. These are the traditional dimensions of disease burden studies and cost of illness studies. The estimated values reveal the colossal negative impact that treatment-resistant depression and major depression with suicide risk have on health and economic resources.

The prevalence of TRD only, MDSR only, and the combined prevalence of the two types of depression were estimated at 79.4 thousand, 52.5 thousand and 11.3 thousand patients, respectively. The disease burden (DALY) due to the disability generated by TRD alone, by MDSR alone, and by the joint prevalence was 25.2 thousand, 21 thousand, and 4.5 thousand, respectively, totaling 50.7 thousand DALY. The disease burden due to premature death by suicide, attributed to TRD and MDSR, was 15.6 thousand DALY. The estimated total disease burden was 66.3 thousand DALY. This figure can be compared with estimates available for other diseases in Portugal. Henriques et al. (44) estimated that ischemic heart disease in Portugal generated 95,413 DALY, which means that TRD and MDSR are responsible for a disease burden that represents ~70% of the ischemic heart disease burden. On the other hand, Gouveia et al. (45) estimated that heart failure in Portugal carried a burden of 21,162 DALY, less than half of the estimated disease burden for TRD and MDSR.

Direct costs of TRD and MDSR were estimated at € 30.8 million, with the most important components being consultations and medication. The estimated indirect costs are much higher than the direct costs. Adding the productivity losses due to the reduction in the level of employment, absenteeism and presenteeism, and the productivity lost due to premature death, a total cost of € 1 billion was calculated. A possible comparison term is given by the costs of asthma in adults in Portugal, estimated by Barbosa et al. (46) at € 386.3 million, 93% of which are direct costs. The direct costs of asthma would thus be almost 12 times higher than those of TRD and MDSR, but the total costs of asthma would only be about 35% of the costs of TRD and MDSR, showing the great indirect costs that these pathologies generate.

The basis for this study was the National Epidemiological Study of Mental Health, that was performed between 2008 and 2009. Despite the NESMH's quality, the lack of more recent data is a limitation, and thus an extrapolation of results to the 2017 population was performed.

Another limitation associated with NESMH is that in this study, the diagnoses were not validated as they were not made by clinicians. However, psychiatric disorders were assessed through comprehensive and fully structured interviews designed by the World Mental Health Surveys Initiative, the World Health Organization, and the Harvard University.

The results obtained did not estimate all consequences of TRD and MDSR on the wellbeing of the Portuguese adult population. A limitation of the present study is that it was not possible to estimate hospitalizations and emergency episodes in private hospitals, as no source of information similar to the Hospital Morbidity Database was available to researchers.

## Conclusions

In addition to the years of life lost and the direct and indirect costs, treatment-resistant depression and major depression with suicide risk have very negative effects in various dimensions relevant to the wellbeing and health of the affected population. These additional dimensions include the impact of depression on the educational outcome, the formation and stability of marital unions, fertility, or even on the quality of parental care (11, 47–49). In the economic area, in addition to the estimated effects on the labor market, there are indications of impact on other areas of financial performance of affected individuals (11).

Although TRD and MDSR represent relatively small direct costs to the health system, they have a significant disease burden and productivity costs on the Portuguese economy and society that are highly relevant, making TRD and MDSR priority areas for obtaining health gains.

## Data availability statement

The data analyzed in this study is subject to the following licenses/restrictions: the data that support the findings of this study are available from World Mental Health Survey Initiative and Health Ministry, but restrictions apply to the availability of these data, which were used under license for the current study, and so are not publicly available. Data are however available from the authors upon reasonable request and with permission at www.hcp.med.harvard.edu/wmh and at www.acss.min-saude.pt.

## Ethics statement

This research did not use human participants and no patient data was presented. National Epidemiological Study of Mental Health was conducted according to all ethical requirements and its ethical procedures are described elsewhere.

## Author contributions

MG, AR, and HC designed the study. MG, RS, AR, GC, AA, HC, and JA contributed with scientific knowledge and data, developed the study, and interpreted the data. MG carried out data analysis. RS written the manuscript. MG, CN-d-S, JA, GC, and AA revised the manuscript. All authors contributed to the article and approved the submitted version.

## Funding

The present work received support from Janssen-Cilag Farmacêutica, Lda, as an Unrestricted Grant. Janssen-Cilag had no role in the execution of the study, including analyses and interpretation of results.

## Conflict of interest

The authors declare that the research was conducted in the absence of any commercial or financial relationships that could be construed as a potential conflict of interest.

## Publisher's note

All claims expressed in this article are solely those of the authors and do not necessarily represent those of their affiliated organizations, or those of the publisher, the editors and the reviewers. Any product that may be evaluated in this article, or claim that may be made by its manufacturer, is not guaranteed or endorsed by the publisher.

## Abbreviations

AF: Attributable Fraction
ATC: Anatomical Therapeutic Chemical
DALY: Disability Adjusted Life Years
HDG: Homogeneous Diagnostic Groups
hmR: Health Market Research
ICD 10-CM, International Classification of Diseases 10: Clinical Modification
ICD 9-CM, International Classification of Diseases 9: Clinical Modification
MDSR: Major Depression with Suicide Risk
NESMH: National Epidemiological Study of Mental Health
RR: Relative Risk
TRD: Treatment-Resistant Depression
WHO: World Health Organization
WMH-CIDI: World Health Organization World Mental Health Composite International Diagnostic Interview
WMHSI: World Mental Health Surveys Initiative
YLD: Years Lived with Disability
YLL: Years of Life Lost.
